# Difference in Mortality Rates by Occupation in Japanese Male Workers Aged 25 to 64 Years from 1980 to 2015

**DOI:** 10.3390/ijerph191811328

**Published:** 2022-09-09

**Authors:** Bibha Dhungel, Tomoe Murakami, Koji Wada, Shunya Ikeda, Stuart Gilmour

**Affiliations:** 1 Graduate School of Public Health, St. Luke’s International University, Tsukiji, Tokyo 104-0044, Japan; 2 Department of Health Policy, National Centre for Child Health and Development, Setagaya, Tokyo 157-8535, Japan; 3 Graduate School of Medicine, International University of Health and Welfare, Akasaka, Tokyo 107-8402, Japan

**Keywords:** mortality, occupational mortality, Japan, managers, suicide, cancer

## Abstract

This study examines the trends in mortality among Japanese working men, across various occupational categories, from 1980 to 2015. A Poisson model of trend, occupational category, and step variable was analysed for eight occupational categories separately, by cause, to explore the trends in mortality. This study found a sharp increase in mortality in the late 1990s, especially among professionals and managers. The overall trends in cancer, ischemic heart disease (IHD), cerebrovascular disease (CVD), and suicide mortality decreased across almost all occupational categories from 1980 to 2015, although there was an increasing trend in cancer of 0.5% among managers. Clerical workers had the greatest relative decrease in mortality rates from cancer (−82.9%), IHD (−81.7%), and CVD (−89.1%). Japan continues to make gains in lowering mortality and extending life expectancy, but its workplace culture must improve to ensure that those working at the heart of the Japanese corporate world can also benefit from Japan’s progress in health. Mortality rates in working-aged Japanese men have been declining. However, similar declines are not evident among managers, for whom the mortality rate is remaining stable or slightly increasing. There is a need to address the needs of managers and improve workplace environments for these workers.

## 1. Introduction

Employment, an important component of socioeconomic status, has been identified as being inversely associated with mortality [1] and is also a fundamental determinant of health [2]. As employed people spend considerable time in the workplace, it becomes an essential part of everyday life. Work-related health hazards are important causes of mortality and morbidity and are avoidable to some extent [3]. Economic crises and other changes in trends in the labour market adversely affect health, health inequalities, and mortality, depending on employment status and occupation [4]. The economic bubble burst in the 1990s, and the global financial crisis of 2008–2009 had a significant impact around the world, including in Japan. In 2008, the unemployment rate in Japan peaked at an all-time post-war high of 5.6%. The proportion of non-regular workers increased gradually, and individual working hours increased among men [5].

Japan introduced universal health insurance coverage in 1961, and under this scheme all occupations had equal opportunities for health promotion [6]. Nonetheless, large health inequalities persist across the occupational categories in Japan [7,8]. Construction and transportation workers work longer hours in Japan compared with other industries [9]. The shift work, cold, heat, noise, carbon dioxide, and stress these workers experience are occupational risk factors for developing cardiovascular disease [10]. Japan has also pioneered the development of the concept of *karoshi*, defined as death from overwork, in recognition of the extremely long and arduous working hours of many staff in ordinary companies in Japan [11]. Given the health risks of long working hours, occupational health hazards should be prevented through effective occupational health policies.

Mortality by occupational class in Japan does not follow a clear social gradient as with its Western counterparts [7,12,13]. Previous studies in Japan have found higher mortality rate ratios among professional and managerial workers aged 30 to 59 years compared with non-managerial workers [14]. Due to ongoing labour changes, it is important to assess mortality by occupation in an appropriate manner. Patterns identified in previous eras may no longer apply, and it is essential to know whether inequalities in mortality have narrowed or widened. A long-term trend analysis of mortality by occupation is essential in order to understand the socioeconomic inequalities in health outcomes. Along with education and income, occupation is closely correlated with socioeconomic status. Proportional mortality ratios by occupation help identify the causes of death that are over-represented in certain occupations [15]. Analysing cause-specific mortalities across occupational groups provides information on how absolute mortality rates differ among occupations. Therefore, this study examines and compares the trends in mortality among Japanese men aged 25 to 64 years across various occupational categories, over 35 years, from 1980 to 2015.

## 2. Materials and Methods

Official studies of occupation-specific vital statistics are conducted every five years in Japan, coinciding with the years of population census [16]. Individual data on occupation-specific mortality were obtained from the Vital Statistics Bureau of the Ministry of Health, Labour and Welfare of Japan. Age- and sex-aggregated occupation-specific population data were obtained from the Japanese Population Census [17]. The death certificates included information on the deceased person’s occupation before death, as provided by a family member, and the underlying cause of death, as filled out by the physician. We analysed data from 1980 to 2015, as we were interested in analysing the trend with equal data-points before and after the economic crisis of the mid-1990s. We analysed the occupation-specific mortality data separately, by cause, for men aged 25 to 64 years. Data on unemployed people and instances of missing values for occupation were removed from the analysis. In Japan, women are more likely to work on a part-time or irregular basis (44.2% of employed women in 2019), and 36% of married women are full-time homemakers [18]. We limited this study to men only so as to be relevant to other studies [8,14], as occupation-specific data on Japanese women have low reliability.

Occupation was categorised as per the classification of the Ministry of Health, Labour and Welfare of Japan into eight groups as follows: professional and technical positions (hereinafter professional); managerial and administrative workers (managers); clerks and office workers (clerical); sales; service; security and transport; agriculture, forestry, and fishery workers (agriculture); and manufacturing, production process, construction, mining, cleaning, and packaging workers (manufacturing). For our analysis, security workers were grouped with transportation workers due to the very low number of people (<3%) in these occupations over the years. Mining, carrying/cleaning, and construction, which were categorised under manufacturing in other years, were categorised separately in the years 1980, 1985, 2010, and 2015 [19,20]. Thus, variable coding was changed in accordance with the year 2005 in order to maintain consistency for all years of the study. Cause of death was classified following the International Classification of Diseases (ICD)-9 for the years before 1990, and ICD-10 for 1995 and later. Further details on ICD codes for each cause can be found in Table S2 (see Supplementary Materials). Analysis was conducted for the four major causes of mortality in the working-age population: ischaemic heart disease, cerebrovascular disease, all cancers, and suicide. We categorised age into ten-year intervals: 25–34 years, 35–44 years, 45–54 years, and 55–64 years.

### Statistical Analysis

Age- and occupation-specific deaths were standardised separately, by cause, using the five-year age-specific population of Japan in 1985 as the standard population, and trends in mortality rates were calculated. A Poisson model of the trend, age category, occupation category, and step variable were analysed separately by cause to explore the trends in mortality across all eight occupational categories. The step term indicates whether the mortality occurred before 1995 or after 2000 and is used to adjust for the change in mortality rates after the economic bubble burst in the late 1990s [14]. It was coded as 0 for the years 1980–1995 and 1 for the years 2000–2015. In the model, the year 1980 was centred to zero, and the consecutive years to 5, 10, etc., up to 35, so that the intercept corresponded to the 1980 mortality rate. A two-way interaction of trend and occupation was used for all four causes of death to analyse the different trends by occupation. A linear combination of trend and occupation variable was used to estimate the occupation-specific change in mortality separately, by cause. Stata IC version 15.1 was used for the analysis.

## 3. Results

Table 1 shows the change in the proportion of occupation categories at five-year timepoints over the years. Manufacturing workers constitute the highest proportion of workers throughout the study period. This proportion, however, decreased over time, as the proportion of service workers, clerks, security workers, and professionals increased. The proportions of managers and of agriculture workers decreased rapidly from 1980, reaching very low proportions by 2015.

**Figure 1:**
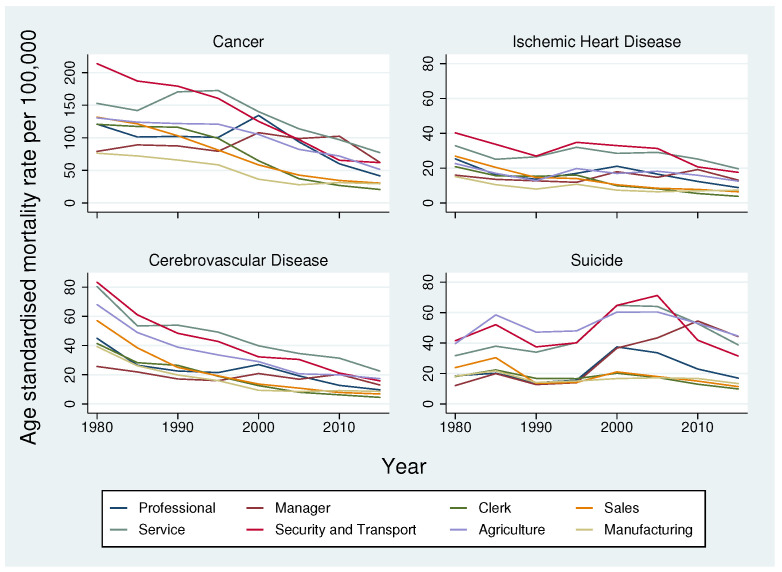
Trend in age-standardised mortality rates per 100,000 by occupation, separately by cause, among Japanese men. Note: Due to a comparatively higher prevalence of cancer mortality compared to the other three causes, a different scale has been used for cancer in the first graph.

Figure 1 shows the trends in age-standardised mortality rates in Japan from 1980 to 2015 for four major causes of mortality: all cancers, ischemic heart disease, cerebrovascular disease, and suicide. The detailed age-standardised rates for all occupations are provided in Table S2 (see Supplementary Materials) Mortality from cancer was almost twice that of other causes of death. The gaps in mortality rates across occupational groups decreased over the years, especially for cancer and cerebrovascular disease. Mortality rates for cancer, ischemic heart disease, and cerebrovascular disease showed a decreasing trend from 1980 to 2015 in all occupational categories except managers and professionals, which showed a sharp increase in mortality in the mid-1990s. However, trends in suicide increased in most occupational categories after the late 1990s; this increase was steepest among service workers, then professionals, and then managers. Compared with other occupations, mortality among manufacturing workers was low for all four causes throughout the study period, while mortality from cancer, ischemic heart disease, and cerebrovascular disease was highest among service workers in 2015.

Table 2 shows the temporal trends by occupational categories for each of the four causes of mortality. Trends in mortality decreased across all occupational categories except for mortality by cancer among managers. The trends in mortality decreased most rapidly among clerical workers for all four causes.

Table 3 shows the absolute and relative differences in mortality rates between 1980 and 2015 for each cause of death across all occupations. There were significant reductions in mortality rates for all cancers, ischemic heart disease, and cerebrovascular disease, while mortality from suicide increased, compared with the rates in 1980, across some occupations. Clerical workers had the greatest relative decrease in mortality rates from cancer (−82.9%), ischemic heart disease (−81.7%), and cerebrovascular disease (−89.1%). The relative overall decrease in mortality rates between 1980 and 2015 for ischemic heart disease and cerebrovascular disease among managers was the smallest. Nonetheless, the relative difference in suicide mortality rate among managers between 1980 and 2015 was very high (+265.3%).

## 4. Discussion

We examined age-standardised temporal trends in mortality by occupation for four leading causes of death—cancer, ischaemic heart disease, cerebrovascular disease, and suicide—from 1980 to 2015, among Japanese men aged 25 to 64 years. The current study shows that the overall trends in mortality from cancer, ischemic heart disease, and cerebrovascular disease decreased across almost all occupational categories from 1980 to 2015, with the highest relative decrease in mortality among clerical and sales workers. In the year 2000, there was a sharp increase in age-standardised mortality rate for all four causes of death among professionals and managers.

Mortality and economic recession show a pro-cyclical relation, where there is a decrease in mortality rates during a recession [21]. A study by the European Union found that rising rates of unemployment during the 2008 recession were associated with a decrease in rates of mortality among individuals aged under 65 years [22], driven by reductions in cardiovascular disease. Mortality rates for professionals and managers rose in the mid- to late-1990s, coinciding with the financial crisis in 1997. Economic stagnation was responsible for changes in the work environment and the employment system, leading to more stressful circumstances for workers [23]. The mean number of annual working hours in Japan decreased, from 2100 h in 1980 to around 1700 h in 2015 [24]. Managers, however, are not subject to work regulations, including the number of working hours, because of ‘white-collar exemption’, Refs. [12,25] which puts them at a high risk of overwork without pay. Studies have also suggested unique patterns of occupation gradient, with strong pressure on high-grade workers in Japan [13]. The number of non-regular workers increased rapidly during the prolonged recession in the early 2000s, replacing numerous workers in regular jobs [5]. To reduce labour costs, the company system was reformed, and the proportion of managers in the labour market decreased from 6.3 per cent in 1990 to 2.9% in 2015. This worker reform probably increased the job responsibilities and working hours of regular employees, especially managers, who oversee and manage non-regular workers. This has potentially led to increased job demands placed on managers compared with other workers.

Studies suggest an association between stress and mortality [26]. Yearly working hours for clerical and sales workers decreased from 2162 to 1970 h between 1980 and 2000. This may partially explain the sharp decrease in mortality rate among clerical workers and the very small increase in suicide rates in service and clerical workers compared with other occupational categories, as also reported in previous studies [8,14]. Following the Lehman shock in 2009, the Japanese annual gross domestic product (GDP) grew by 4.2% in 2010, after shrinking by 5.4% in 2009. The employment situation also began to recover. The proportion of the workforce made up of part-time workers reached its highest percentage (18%) in 2015 [27]. Dispatched employees work under indirect employment conditions, as they work for a temping agency and take up a post in another company through a third party, in line with the instructions of the agency. The manufacturing sector comprises the highest proportion of these dispatched workers [2], and 28.3% of the total foreign labour workforce in Japan are primarily engaged as dispatched labourers [28]. With the reform of the Japanese employment system, there has been a decrease in the proportion of dispatched employees, especially in the manufacturing sector; this figure started rising again in 2013. Japan’s employment availability improved to reach its best in 23 years, and in 2015 the unemployment rate fell to its lowest level in 18 years. Economic fluctuation is associated with both widening and narrowing inequalities in mortality [29]. The improving economic conditions in Japan after 2010 may have contributed to the decreasing occupation-specific mortality trends after 2010.

The Cancer Control Act was introduced in Japan in 2006 to reduce cancer mortality through the standardisation of prevention and treatment measures [30]. With the increasing use of advanced medical technologies such as computed tomography (CT) and PET scans, diseases such as cancer, which is the leading cause of death in Japan, are increasingly detected at an early and more treatable stage [31]. Increased access to advanced screening and treatment could be one reason why mortality from most diseases, including cancer, is decreasing sharply. However, our study findings also suggest an increasing trend in cancer mortality among managers. Tanaka et al. found that administrative and managerial workers in Japan have a higher mortality risk compared with sales workers [32]. A study in China found that managers are more likely to smoke [33]. The increasing workload after the economic crisis in Japan could have had a significant impact on psychological stress among managers, potentially resulting in increased smoking and alcohol consumption, unhealthy eating, and poor exercise habits, resulting in an increased incidence of cancer. Managers in Japan are likely to be supported by their company—or even required—to undergo routine health check-ups, and thus should have the best possibility of being diagnosed early when cancer does occur. High rates of cancer mortality in these professional groups raise concern over the health screening system in Japan and indicate a need to reform this system to ensure it works for those in need. Regular health check-ups, better follow up, improved coordination of screening and treatment services, and better workplace support for those identified as being at risk for cancer should be implemented.

An increased use of antihypertensive drugs under universal health insurance coverage has contributed to long-term reductions in stroke and cardiovascular mortality in Japan [6]. Full-body check-ups such as *Ningen dock* are believed to be helpful in the primary prevention of cerebrovascular and cardiovascular disease through post-check-up consultation involving the control of risk factors such as obesity and hypertension. Our study found the highest cancer, cerebrovascular disease, and ischemic heart disease mortality rates in 2015 to be among service workers. This could be attributed to the higher prevalence of riskier behaviours such as smoking, excessive alcohol consumption, physical inactivity, and poor dietary habits among service workers, compared with professionals [34]. The proportion of service workers increased from 3.5% in 1980 to 5.4% in 2015. Studies have shown growing health inequalities across social classes; [35] however, our findings suggest that the gaps in mortality across occupational categories for cancer and cerebrovascular disease have decreased over the years. The downward trend in occupational disparities in mortality rates continued until the early 1990s, possibly attributable to the narrowing of the income gap observed in Japan in the late 1980s, during the financial bubble. However, income inequalities increased in the mid-2000s, thus increasing health disparities in recent years, despite decreasing trends in mortality [13].

A previous study has shown a countercyclical trend of suicide mortality in relation to economic fluctuation in Japan [36]. Older individuals who lost their jobs during the recession had an increased risk of mortality, [37] especially from suicide [22]. Growing unemployment rates during the recession were the probable causal determinants of many, but not all, cases of suicide, [38] especially among working-age men [39]. A one-percentage-point increase in the unemployment rate is associated with a decrease in all-cause mortality, but with a very high increase in suicide mortality [22]. Suicide rates increased only among managers in the year 2010, when the national unemployment rate was very high, while they decreased in all other occupational categories. Since 2014, the number of regular workers has been increasing. Similarly, the rate of unemployment was 2.4% in 2018, the lowest recorded in 26 years, thus contributing to the decrease in suicide mortality. Suicide rates overall have been falling in Japan, [40] suggesting that this inequality in suicide mortality is concentrated among a few occupational groups, and that further efforts are needed to ensure that managers and professionals can benefit from Japan’s overall success in reducing this cause of death.

This study has some limitations. The retirement age in Japan has recently been changed from 60 to 65 years. People who remained in employment after this age in 1980 may be different from those who remained in employment in recent years, when working after 60 years became much more common. This study did not adjust for other potentially confounding factors affecting mortality, such as occupational exposures and duration of employment. There is a possibility of misclassification of occupation, as the data were obtained from the families of the deceased. The reported occupation could be the last or longest occupation, not necessarily reflecting employment or unemployment status immediately before death. Furthermore, estimating mortality rates using both ICD-9 and ICD-10 may have affected the results. It may be necessary to consider the ICD classification comparability ratios in order to interpret the obtained mortality rates. This study focused on mortality among men; further studies are required to explore inequalities in mortality among women.

## 5. Conclusions

The mortality rates in working-aged Japanese men declined between 1980 and 2015. However, similar declines were not evident among managers, who are not subject to work hour regulations. Among this group, the mortality rate increased in the mid-1990s, and remained stable or slightly increased thereafter. There is a need to address the needs of managers and improve workplace environments for these workers. Japan continues to improve its mortality rates and extend its life expectancy, but its workplace culture must improve to ensure that those working in the heart of the Japanese corporate world can benefit equally from the nation’s progress in health.

## Figures and Tables

**Table 1:** Proportion of population among Japanese men of working age by occupation from 1980 to 2015.

Year	1980 N = 29,757,359 (%)	1985 N = 30,955,331 (%)	1990 N = 31,770,983 (%)	1995 N = 32,608,802 (%)	2000 N = 32,316,342 (%)	2005 N = 31,582,583 (%)	2010 N = 30,280,614 (%)	2015 N = 27,744,837 (%)
Managers	2,210,783 (7.4)	1,868,101 (6.0)	1,998,511 (6.3)	2,066,172 (6.3)	1,305,093 (4.0)	1,031,316 (3.3)	905,915 (3.0)	803,754 (2.9)
Professional	2,306,830 (7.8)	3,143,412 (10.2)	3,637,515 (11.4)	3,991,077 (12.2)	4,221,683 (13.1)	3,950,815 (12.5)	4,058,048 (13.4)	4,236,493 (15.3)
Clerk	3,637,048 (12.2)	3,857,022 (12.5)	3,895,784 (12.3)	3,906,006 (12.0)	4,077,310 (12.6)	4,093,124 (13.0)	4,029,507 (13.3)	4,023,221 (14.5)
Sales	4,132,015 (13.9)	4,509,884 (14.6)	4,794,455 (15.1)	5,044,836 (15.5)	5,159,661 (16.0)	4,716,064 (14.9)	3,889,327 (12.8)	3,400,906 (12.3)
Service	1,027,910 (3.5)	1,123,385 (3.6)	1,202,319 (3.8)	1,270,668 (3.9)	1,381,504 (4.3)	1,441,522 (4.6)	1,570,707 (5.2)	1,493,970 (5.4)
Security	567,438 (1.9)	615,053 (2.0)	660,161 (2.1)	706,462 (2.2)	787,325 (2.4)	832,148 (2.6)	829,146 (2.7)	792,305 (2.9)
Agriculture	2,379,666 (8.0)	2,112,513 (6.8)	1,615,756 (5.1)	1,199,620 (3.7)	899,881 (2.8)	823,066 (2.6)	759,012 (2.5)	649,351 (2.3)
Transport	2,072,133 (7.0)	1,997,137 (6.5)	1,984,890 (6.2)	2,020,393 (6.2)	1,957,847 (6.1)	1,794,551 (5.7)	1,780,306 (5.9)	1,578,203 (5.7)
Manufacturing	10,682,007 (35.9)	10,644,436 (34.4)	10,985,461 (34.6)	10,945,330 (33.6)	10,762,241 (33.3)	10,451,026 (33.1)	8,797,698 (29.1)	8,168,791 (29.4)

**Table 2:** Temporal trends by occupational categories for major causes of mortality from 1980 to 2015.

Occupational Category	Cancer		Ischaemic Heart Disease		Cerebrovascular Disease		Suicide	
	RR ^†^	CI ^‡^	RR ^†^	CI ^‡^	RR ^†^	CI ^‡^	RR ^†^	CI ^‡^
Manager	1.005	(1.003–1.006)	0.994	(0.990–0.998)	0.978	(0.975–0.982)	0.988	(0.982–0.995)
Professional	0.982	(0.981–0.984)	0.980	(0.976–0.983)	0.966	(0.963–0.968)	0.960	(0.956–0.964)
Clerk	0.953	(0.952–0.954)	0.954	(0.950–0.957)	0.937	(0.934–0.939)	0.968	(0.963–0.972)
Sales	0.961	(0.960–0.962)	0.958	(0.955–0.962)	0.937	(0.934–0.940)	0.958	(0.954–0.963)
Service	0.985	(0.983–0.987)	0.988	(0.984–0.991)	0.967	(0.964–0.970)	0.982	(0.977–0.987)
Security and Transport	0.969	(0.967–0.970)	0.981	(0.977–0.984)	0.957	(0.954–0.960)	0.967	(0.962–0.972)
Agriculture	0.989	(0.987–0.990)	0.984	(0.980–0.988)	0.954	(0.951–0.957)	0.999	(0.994–1.004)
Manufacturing	0.971	(0.970–0.972)	0.975	(0.972–0.978)	0.948	(0.945–0.950)	0.982	(0.979–0.985)
All Occupations	0.975	(0.974–0.976)	0.976	(0.974–0.978)	0.951	(0.949–0.952)	0.974	(0.972–0.975)

^†^ Rate ratio per five years, adjusting for trend, age, occupational category, step term, and interaction of occupational category and trend; ^‡^ Confidence interval. Rate ratios are calculated from linear combinations of the coefficients of trend and occupation variables separately for each cause, incorporating covariance among coefficients, to ensure the correct calculation of standard errors.

**Table 3:** Age-standardised absolute and relative differences in mortality rates between 1980 and 2015, by cause.

Occupational Categories	All Cancer				Ischemic Heart Disease				Cerebrovascular Disease				Suicide			
	1980	2015	Difference ^†^	% ^‡^	1980	2015	Difference ^†^	% ^‡^	1980	2015	Difference ^†^	% ^‡^	1980	2015	Difference ^†^	% ^‡^
Managers	79.1	62.3	−16.8	−21.2	16.0	13.1	−2.9	−18.1	25.7	13.0	−12.7	−49.4	12.1	44.2	32.1	265.3
Professional	121.0	41.6	−79.4	−65.6	25.3	8.8	−16.5	−65.2	44.9	9.7	−35.2	−78.4	18.5	17.0	−1.5	−8.1
Clerk	120.7	20.7	−100.0	−82.9	20.8	3.8	−17.0	−81.7	41.4	4.5	−36.9	−89.1	18.0	9.9	−8.1	−45.0
Sales	131.6	30.5	−101.1	−76.8	26.9	6.4	−20.5	−76.2	57.1	6.9	−50.2	−87.9	23.9	11.4	−12.5	−52.3
Service	152.8	77.2	−75.6	−49.5	32.9	19.7	−13.2	−40.1	80.5	22.5	−58.0	−72.0	31.7	38.8	7.1	22.4
Security	213.8	62.0	−151.8	−71.0	40.3	17.6	−22.7	−56.3	83.4	15.8	−67.6	−81.1	41.5	31.5	−10.0	−24.1
Agriculture	130.5	51.6	−78.9	−60.5	22.6	12.5	−10.1	−44.7	68.1	17.3	−50.8	−74.6	39.6	44.6	5.0	12.6
Manufacturing	76.3	29.5	−46.8	−61.3	15.2	7.3	−7.9	−52.0	39.3	8.6	−30.7	−78.1	18.3	13.4	−4.9	−26.81
All Occupations	141.6	47.5	−94.2	−66.5	19.9	8.0	−11.9	−59.7	46.0	8.9	−37.1	−80.7	20.6	16.0	−4.7	−22.7

^†^ Absolute difference in age-standardised mortality rate per 100,000 between 1980 and 2015; ^‡^ difference between age-standardised rates of 2015 and 1980 expressed as percentage of the 1980 rate.

## Data Availability

The data that support the findings of this study are available from the Japanese Ministry of Health, Labour and Welfare; restrictions apply to the availability of these data, however, and they are not publicly available.

## Supplementary Materials

The following supporting information can be downloaded at https://www.mdpi.com/article/10.3390/ijerph191811328/s1: Table S1: ICD codes used for categorising cause of mortality by year and ICD era; Table S2: Age-standardised mortality rates per 100,000 by cause, for various occupational categories from 1980 to 2015.

## References

[1] Davey Smith G., Hart C., Hole D., MacKinnon P., Gillis C., Watt G., Blane D., Hawthorne V. (1998). Education and Occupational Social Class: Which Is the More Important Indicator of Mortality Risk?. J. Epidemiol. Community Health.

[2] Asao Y. Overview of Non-Regular Employment in Japan. In: Non-Regular Employment—Issues and Challenges Common to the Major Developed Countries: 2011 JILPT Seminar on Non-Regular Employment. https://www.jil.go.jp/english/reports/documents/jilpt-reports/no.10.pdf.

[3] Tsuchiya A., Wada K., Morikane K., Yoshikawa T., Hosomi Y., Dhungel B., Kunishima H. (2022). Characteristics of Needlestick and Sharps Injuries of the Hands in the Operating Room among Orthopedic Surgeons in Japan. Ind. Health.

[4] Glonti K., Gordeev V.S., Goryakin Y., Reeves A., Stuckler D., McKee M., Roberts B. (2015). A Systematic Review on Health Resilience to Economic Crises. PLoS ONE.

[5] Genda Y., Kuroda S., Ohta S. (2015). Does Downsizing Take a Toll on Retained Staff? An Analysis of Increased Working Hours in the Early 2000s in Japan. J. Jpn. Int. Econ..

[6] Ikeda N., Saito E., Kondo N., Inoue M., Ikeda S., Satoh T., Wada K., Stickley A., Katanoda K., Mizoue T. (2011). What Has Made the Population of Japan Healthy?. Lancet.

[7] Dhungel B., Takagi K., Acharya S., Wada K., Gilmour S. (2022). Changes in Cause-Specific Mortality Trends across Occupations in Working-Age Japanese Women from 1980 to 2015: A Cross-Sectional Analysis. BMC Womens Health.

[8] Wada K., Gilmour S. (2016). Inequality in Mortality by Occupation Related to Economic Crisis from 1980 to 2010 among Working-Age Japanese Males. Sci. Rep..

[9] Ministry of Internal Affairs and Communication Monthly Working Hours by Industry. http://www.stat.go.jp/data/nihon/16.html.

[10] Shalat S.L., Robson M.G., Mohr S.N. (2005). Agricultural Workers.

[11] Wada K., Eguchi H., Prieto-Merino D. (2016). Differences in Stroke and Ischemic Heart Disease Mortality by Occupation and Industry among Japanese Working-Aged Men. SSM Popul. Health.

[12] Dhungel B., Murakami T., Wada K., Gilmour S. (2021). Mortality Risks among Blue- and White-collar Workers: A Time Series Study among Japanese Men Aged 25–64 Years from 1980 to 2015. J. Occup. Health.

[13] Kagamimori S., Gaina A., Nasermoaddeli A. (2009). Socioeconomic Status and Health in the Japanese Population. Soc. Sci. Med..

[14] Wada K., Kondo N., Gilmour S., Ichida Y., Fujino Y., Satoh T., Shibuya K. (2012). Trends in Cause Specific Mortality across Occupations in Japanese Men of Working Age during Period of Economic Stagnation, 1980–2005: Retrospective Cohort Study. BMJ.

[15] Coggon D., Harris E.C., Brown T., Rice S., Palmer K.T. (2010). Work-Related Mortality in England and Wales, 1979–2000. Occup. Environ. Med..

[16] Ministry of Health Labour and Welfare Vital Statistics of Japan 1980–2015. https://www.mhlw.go.jp/english/database/db-hw/index.html.

[17] Statistics Bureau of Japan Population Census 1980–2015. http://www.stat.go.jp/english/data/kokusei/index.html.

[18] Ministry of Internal Affairs and Communications Labour Force Survey 2019: Population Aged 15 Years Old and over by Labour Force Status, Status in Employment, Type of Employment. https://www.e-stat.go.jp/en/stat-search/files?page=1&layout=datalist&toukei=00200531&tstat=000000110001&cycle=7&year=20190&month=0&tclass1=000001040276&tclass2=000001040283&tclass3=000001040284&stat_infid=000031905238&result_back=1&tclass4val=0.

[19] Ministry of Internal Affairs and Communications Japan Standard Occupational Classification (Rev. 5th, December 2009). https://www.soumu.go.jp/english/dgpp_ss/seido/shokgyou/co09-1.htm.

[20] Ministry of Health Labour and Welfare Occupational Classification (Revision 4th). https://www.jil.go.jp/institute/seika/shokugyo/bunrui/index.html.

[21] Margerison-Zilko C., Goldman-Mellor S., Falconi A., Downing J. (2016). Health Impacts of the Great Recession: A Critical Review. Curr. Epidemiol. Rep..

[22] Toffolutti V., Suhrcke M. (2014). Assessing the Short Term Health Impact of the Great Recession in the European Union: A Cross-Country Panel Analysis. Prev. Med..

[23] Tachibanaki T. (2005). Confronting Income Inequality in Japan: A Comparative Analysis of Causes, Consequences, and Reform.

[24] The Japan Institute for Labour Policy and Training Japanese Working Life Profile 2016/2017. https://www.jil.go.jp/english/jwl/2016-2017/all.pdf.

[25] Kuroda S., Yamamoto I. How Are Hours Worked and Wages Affected by Labor Regulations? The White-Collar Exemption and “name-Only Managers” in Japan. https://jww.iss.u-tokyo.ac.jp/publishments/dp/dpf/pdf/f-147.pdf.

[26] Darr W., Johns G. (2008). Work Strain, Health, and Absenteeism: A Meta-Analysis. J. Occup. Health Psychol..

[27] The Japan Institute of Labour Policy and Training (2016). Labor Situation in Japan and Its Analysis: General Overview 2015/2016.

[28] Ministry of Health Labour and Welfare of Japan Results of the Report on the Employment Conditions of Foreigners. https://www.mhlw.go.jp/english/database/db-l/foreigners04/index.html.

[29] Chen G. (2014). Association between Economic Fluctuations and Road Mortality in OECD Countries. Eur. J. Public Health.

[30] Ministry of Health Labour and Welfare of Japan (2012). Health and Medical Services.

[31] Miho Kawasaki Ningen Dock: Japanese-Style Health Examinations. http://japan-product.com/ningen-dock/.

[32] Tanaka H., Tanaka T., Wada K. (2020). Mortality by Occupation and Industry among Japanese Men in the 2015 Fiscal Year. Environ. Health Prev. Med..

[33] Wang Q., Shen J.J., Sotero M., Li C.A., Hou Z. (2018). Income, Occupation and Education: Are They Related to Smoking Behaviors in China?. PLoS ONE.

[34] Fukuda Y., Nakamura K., Takano T. (2005). Accumulation of Health Risk Behaviours Is Associated with Lower Socioeconomic Status and Women’s Urban Residence: A Multilevel Analysis in Japan. BMC Public Health.

[35] Mackenbach J.P., Bos V., Andersen O., Cardano M., Costa G., Harding S., Reid A., Hemström O., Valkonen T., Kunst A.E. (2003). Widening Socioeconomic Inequalities in Mortality in Six Western European Countries. Int. J. Epidemiol..

[36] Granados J.A.T. (2008). Macroeconomic Fluctuations and Mortality in Postwar Japan. Demography.

[37] Noelke C., Beckfield J. (2014). Recessions, Job Loss, and Mortality Among Older US Adults. Am. J. Public Health.

[38] DeFina R., Hannon L. (2015). The Changing Relationship Between Unemployment and Suicide. Suicide Life Threat. Behav..

[39] Phillips J.A., Nugent C.N. (2014). Suicide and the Great Recession of 2007–2009: The Role of Economic Factors in the 50 U.S. States. Soc. Sci. Med..

[40] Dhungel B., Sugai M.K., Gilmour S. (2019). Trends in Suicide Mortality by Method from 1979 to 2015 in Japan. Int. J. Environ. Res. Public Health.

