# Supplementary material for: Difference in Mortality Rates by Occupation in Japanese Male Workers Aged 25 to 64 Years from 1980 to 2015
Source: Int J Environ Res Public Health. 2022 Sep 9;19(18):11328. doi: 10.3390/ijerph191811328 (PMC9517138; doi:10.3390/ijerph191811328)
Supplement: Supplementary file 1 [file ijerph-19-11328-s001.zip › ijerph-1844572-supplementary.pdf]

Table S1. ICD codes used for categorizing cause of mortality by year and ICD era

Years	ICD version	ICD codes			
		Cancer	Ischemic Heart Disease	Cerebrovascular Disease	Suicide
1980 – 1990	9	140–239	410–414	430–438	E950–E959
1995 – 2015	10	C00–D48	I20–I25	I60–I69	X60–X84

Table S2. Age-standardised mortality rates per 100,000 by cause for various occupational categories from 1980 to 2015

Cause/Occupation	1980	1985	1990	1995	2000	2005	2010	2015
**All cancer**								
Professional	121.0	101.2	102.2	100.6	134.4	93.7	60.1	41.6
Manager	79.1	89.2	87.5	79.3	107.9	98.9	102.4	62.3
Clerk	120.7	117.4	116.2	99.1	64.7	37.2	26.8	20.7
Sales	131.6	121.4	103.1	81.2	58.4	42.7	34.5	30.5
Service	152.8	141.8	170.5	172.6	140.1	113.8	97.0	77.2
Security and transport	213.8	187.1	179.2	160.5	125.2	97.2	65.5	62.0
Agriculture	130.5	124.0	121.8	120.9	105.1	82.2	72.0	51.6
Manufacturing	76.3	72.2	65.9	58.5	36.4	27.9	31.3	29.5
**Ischaemic Heart Disease**								
Professional	25.3	16.3	13.6	17.0	21.1	16.6	12.4	8.8
Manager	16.0	13.6	12.7	11.9	17.9	14.7	19.2	13.1
Clerk	20.8	15.6	15.3	16.0	9.9	8.1	5.4	3.8
Sales	26.9	20.4	14.6	14.0	10.5	8.5	7.8	6.4
Service	32.9	25.1	26.5	32.0	28.5	29.1	25.2	19.7
Security and transport	40.3	33.7	26.9	34.8	32.9	31.3	20.7	17.6
Agriculture	22.6	17.0	12.8	19.8	17.0	18.2	15.9	12.5
Manufacturing	15.2	10.5	8.0	10.7	7.3	6.4	7.0	7.3
**Cerebrovascular Disease**								
Professional	44.9	26.5	22.6	21.5	27.0	19.2	12.7	9.7
Manager	25.7	21.9	17.1	15.9	20.8	16.9	20.3	13.0
Clerk	41.4	28.2	26.4	19.0	12.6	8.0	6.2	4.5
Sales	57.1	38.4	25.0	19.2	13.6	10.8	7.8	6.9
Service	80.5	53.4	53.9	49.2	39.8	34.5	31.3	22.5
Security and transport	83.4	61.0	48.3	42.8	32.2	30.5	21.2	15.8
Agriculture	68.1	48.9	38.9	33.6	29.0	20.6	19.9	17.3
Manufacturing	39.3	26.1	19.8	16.0	9.4	8.5	9.1	8.6
**Suicide**								
Professional	18.5	20.2	14.0	15.6	37.5	33.6	23.0	17.0
Manager	12.1	19.9	12.7	13.9	36.7	43.4	54.5	44.2
Clerk	18.0	22.4	16.8	16.6	20.2	17.4	13.0	9.9
Sales	23.9	30.4	13.5	14.2	21.1	18.0	15.0	11.4
Service	31.7	37.9	34.0	40.3	64.8	64.1	52.8	38.8
Security and transport	41.5	52.1	37.5	40.2	64.7	71.3	41.9	31.5
Agriculture	39.6	58.5	47.2	48.0	60.3	60.4	53.0	44.6
Manufacturing	18.3	21.7	14.1	15.1	16.7	17.2	16.7	13.4
